# Metabarcoding Analysis of Bacterial Communities Associated with Media Grow Bed Zones in an Aquaponic System

**DOI:** 10.1155/2020/8884070

**Published:** 2020-10-01

**Authors:** Nasser Kasozi, Horst Kaiser, Brendan Wilhelmi

**Affiliations:** ^1^Department of Biochemistry and Microbiology, Rhodes University, P.O. Box 94, Grahamstown 6140, South Africa; ^2^Animal Resources Research Programme, Abi Zonal Agricultural Research and Development Institute, National Agricultural Research Organisation, P.O. Box 219, Arua, Uganda; ^3^Department of Ichthyology and Fisheries Science, Rhodes University, P.O. Box 94, Grahamstown 6140, South Africa

## Abstract

The development of environmentally sustainable plant and fish production in aquaponic systems requires a complete understanding of the systems' biological components. In order to better understand the role of microorganisms in this association, we studied the bacterial communities in the dry, root, and mineralized zones of a flood-and-drain media bed aquaponic system. Bacterial communities were characterized using metabarcoding of the V3-V4 16S rRNA regions obtained from paired-end Illumina MiSeq reads. Proteobacteria, Actinobacteria, and Bacteroidetes accounted for more than 90% of the total community in the dry zone and the effluent water. These phyla also accounted for more than 68% of the total community in the root and mineralized zones. The genera *Massilia*, *Mucilaginibacter*, *Mizugakiibacter*, and *Rhodoluna* were most dominant in the dry, root, and mineralized zones and in the effluent water, respectively. The number of shared operational taxonomic units (OTUs) for the three zones was 241, representing 7.15% of the total observed OTUs. The number of unique OTUs in samples from dry zone, root zone, mineralized zone, and effluent water was 485, 638, 445, and 383, respectively. The samples from the root zone harbored more diverse communities than either the dry or mineralized zones. This study is the first to report on the bacterial community within the zones of a flood-and-drain media bed. Thus, this information will potentially accelerate studies on other microbial communities involved in the bioconversion of nitrogen compounds and mineralization within these types of aquaponic systems.

## 1. Introduction

Aquaponics is an integrated farming concept that combines the production of fish and hydroponic plants in systems that rely on microbial activity to improve water quality and provide nutrients to plants, in either single process loop (coupled) or two-loop systems (decoupled) [1, 2]. The accumulation of uneaten food, fish feces, and organic and inorganic nitrogenous compounds in the system provides an ecosystem for microbial activity and development resulting in their conversion to plant nutrients [3]. During nitrification, ammonia, the main form of inorganic nitrogen excreted by fish, is oxidized either directly to nitrate [4] or via nitrite to nitrate, the latter being less toxic to fish, while plants can utilize it for growth [5, 6]. An important feature of aquaponics is the reliance on microbial activity. Both autotrophic and heterotrophic bacteria occur in aquaponic systems [6, 7]. They play a role in rhizosphere remediation, the control of abiotic stressors, and the protection of plants from pathogens [8].

Media-based growth systems are frequently used because of their simplicity and reliability [3, 9]. Media beds are designed to flood and drain or to operate as constant-flow systems [10]. Flood-and-drain cycles enable the hydroponic media bed to acquire atmospheric air which can lead to aerobic conditions in parts of the media bed [10]. Consequently, there are distinct vertically differentiated zones in media bed of flood-and-drain systems [3]. The media bed provides stability for root growth, and plants with extensive root systems typically adapt well to this environment [11]. Flood-and-drain aquaponic systems also provide a high surface area habitat for beneficial bacteria, as well as being a filter system for particulate suspended matter.

An international survey showed that 86% of aquaponic farmers used media-based systems, while 35% of respondents used a combination of media bed and a raft growing technique [12]. Mchunu et al. [13] reported that 96% of survey respondents adopted media-based aquaponics in South Africa with the flood-and-drain media bed culture being the most frequently used design. There is a paucity of research on microbial diversity in aquaponic systems, particularly on the interactions between microbiota across vertical zones of the media bed that can function as micro-ecosystems [3]. As the media bed provides a niche for diverse populations of micro- and macro-organisms, knowledge of bacterial diversity and spatial distribution of microbes in a flood-and-drain media bed will contribute to our understanding of microbial community dynamics. This fundamental knowledge should have benefits for applied research that focusses on enhancing water quality and the growth and health of fish and plants.

Metabarcoding using the 16S rRNA marker has been frequently used when studying microbial communities [14, 15]. Next-generation sequencing (NGS) metabarcoding has been effectively used in the evaluation of microbial communities of many environmental samples and for the analysis of communities of microbes that cannot be easily cultured on selected media [6, 14, 15]. This technique has been applied to investigate the microbial diversity in different compartments and substrates of aquaponic systems such as plant roots, the system's sump, or the biological filter [7, 16, 17], but not when comparing microbial community diversity in vertically arranged zones of the media bed.

For practical reasons, such as the demand on time and computational and monetary constraints, environmental microbiologists have reported findings related to microbial diversity using limited numbers of experimental units [18, 19]. To contribute to the database that has been generated in studies, in which data from one point in time and from one aquaponic system were generated [7, 17], the aim of this study was to describe the diversity of bacterial communities in a flood-and-drain media bed system and to suggest biological processes that may occur in the vertical zones of this environment.

## 2. Materials and Methods

### 2.1. Ethics Statement

The study was conducted in accordance with the ethical guidelines for the use of animals in research and was approved by the Animal Research Ethics Committee (AREC) of the Rhodes University, South Africa (RU-AREC references: 29102018 and 2019-1145-2120).

### 2.2. Description of the Media Bed System

The samples analyzed in this study were collected from a mature aquaponic system (Practical Aquaponics Farm in Salem, South Africa). The system consisted of fish tanks stocked with Mozambique tilapia, *Oreochromis mossambicus*, two sumps, and gravel beds connected to deep water culture units. Water from the fish tank continuously flowed to the sump. The water, containing organic waste solids and ammonia, was then fed to the gravel flood-and-drain media beds. Water from the media beds drained to floating raft beds. Both the gravel and floating raft beds served as mechanical and biological filters. The water was then returned to the fish tank from where the cycle was repeated. Media beds were made from food-grade plastic materials. The depth and width of the media bed were 35 cm and 120 cm, respectively. Each of the media bed carried approximately 0.5 m^3^ of wet gravel or 24 m^2^ of the biological surface area. Gravel stones measuring approximately 19 mm were used as media within the grow bed. The number of flood cycles was maintained at four cycles per hour and during the time of sampling, the average pH of the effluent water was 6.8.

### 2.3. Sediment and Water Sampling

**Figure 1:**
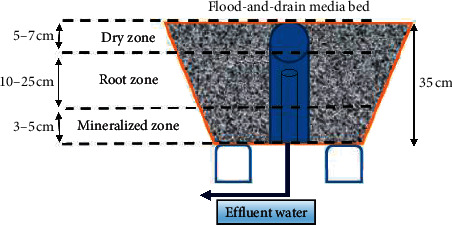
A schematic indicating the three zones of flood-and-drain media bed. The different zones of the flood-and-drain media bed indicated in the layout is described according to Somerville et al. [3].

Gravel stone samples were collected using a sterilized stainless-steel shovel. Triplicate samples (200 g each) from the top dry zone (5–7 cm depth), root zone (10–25 cm depth), and the bottom mineralized zone (3–5 cm depth) were collected from a flood-and-drain media bed (Figure 1). In addition, 2 L of water was collected in sterile Pyrex bottles from the grow bed through the outlet pipe. The triplicate samples were taken to reflect a representation of the microbial population present in each zone. The samples were transferred to sterile plastic bags, and excess water was removed. All samples were transported on ice to the laboratory within 2 h, kept at 2°C, and prepared for analyses the following day.

### 2.4. DNA Extraction

In order to ensure the harvest of a maximum quantity of bacteria, pooled gravel samples for each zone were transferred to a 250 mL sterile beaker containing 150 mL of sterilized water. After vigorous stirring for 10 min, 100 mL of water from each beaker was filtered through 0.2 *μ*m filters (Supor® Membrane disc filters, PALL Life Sciences, USA) with a vacuum pump (Rocker Model 801, vacuum pump 167801-22, Taiwan). This procedure was repeated three times for each sample. Water samples were also filtered through 0.2 *μ*m filters with the vacuum pump. All filters corresponding to different samples were transferred to ZR Bashing Bead™ lysis tubes comprising 0.5 mm beads. DNA was extracted from microbial cells associated with filter materials using a ZymoBIOMICS™ DNA Miniprep Kit (USA) according to the manufacturer's instructions. The extracted DNA concentration was measured using a NanoDrop™ 2000 (Thermo Fisher Scientific, USA), run on 1% agarose gel electrophoresis, and then visualized using a ChemiDoc™ XRS+ (Bio-Rad, Hercules, CA, USA).

### 2.5. PCR Amplification and Purification

**Figure 2:**
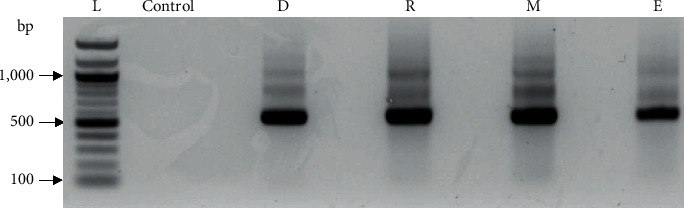
Agarose gel electrophoresis of the PCR products amplified from the samples collected from different zones of flood-and-drain media bed. Lane L shows the 1,000 bp ladder, followed by a negative control. Lanes D, R, M, and E are the amplification products from dry zone, root zone, mineralized zone, and effluent water, respectively.

Amplification of the variable region V3-V4 of the 16S rRNA was performed using a universal primer set 16Sa-F (5′-TCG TCG GCA GCG TCA GAT GTG TAT AAG AGA CAG CAG CAG CCG CGG TAA- 3′) and 16Sa-R (5′-GTC TCG TGG GCT CGG AGA TGT GTA TAA GAG ACA GGT AAG GTT CYT CGC GT- 3′). Each reaction mixture contained 50 ng of template DNA, 0.3 *μ*M of each oligonucleotide primer, 0.3 mM of dNTP mix, 1 × reaction buffer, 2.5 mM of MgCl_2_, and 1 unit of Accupol™ DNA polymerase, and PCR-grade water was added to a total of 25 *μ*L. A control, in which nuclease-free water was added instead of DNA, was included in the samples used for PCR. The samples were amplified using a T100™ Thermal Cycler (Bio-Rad Laboratories, Hercules, CA, USA) with the following conditions: initial denaturation at 98°C for 5 min; 7 cycles of 98°C for 45 s, 45°C for 30 s, and 72°C for 1 min followed by 18 cycles at 98°C for 30 s, 50°C for 30 s, and 72°C for 1 min. A final extension was done at 72°C for 5 min. Electrophoresis was performed for 45 min at 100 V. PCR products were visualized on a 1% agarose gel under a ChemiDoc™ XRS+ (Bio-Rad, Hercules, CA, USA) (Figure 2). The expected size of amplicons was approximately 550 bp, which were excised from the gel and purified using the Zymoclean™ Gel DNA recovery Kit (Zymo Research) according to kit instructions. The gel purification was confirmed by electrophoresis on a 1% agarose gel under a ChemiDoc™ XRS+ system (Bio-Rad, Hercules, CA, USA). A final volume of 15 *μ*L of purified amplicon products was obtained. After purification, DNA was quantified using the PicoGreen assay (Invitrogen) and the quality was checked using a bioanalyzer (Agilent). The Illumina MiSeq integrated next-generation sequencer (Illumina® Inc., USA) and a MiSeq Reagent Kit v3 (600 cycles) (Illumina® Inc. USA) were used to sequence the prepared amplicon libraries. The Nextera XT adaptors were used to multiplex the amplicon libraries before loading onto the MiSeq and sequencing.

### 2.6. Data Curation and Analyses

The sequence FASTQ files from the Illumina MiSeq were analyzed using Mothur platform version 1.41.3 release [20]. Dataset curation included removal of reads shorter than 100 bases, reads longer than 500 bases, and those with ambiguous nucleotides. Chimeric sequences were removed using the VSEARCH [21] command within Mothur. Subsequently, unique reads were checked for chimeric sequences followed by their removal from the datasets. Classification of the sequence reads was done using Naïve Bayesian classifier against the Silva bacterial database (release version 132) and plotted as a percentage of total reads per sample. Nonbacterial, chloroplast, and mitochondrial operational taxonomic units (OTUs) were considered as contaminating sequences and removed prior to downstream analysis. All OTUs were clustered at a cutoff of 0.03. The taxonomical classification was performed to genus level (Supplementary Table S1). Alpha diversity metrics (InvSimpson, Chao1, Shannon, and Good's coverage index) were calculated using the Mothur platform. The analysis of the common and unique OTUs was conducted to investigate the media bed bacterial communities through a Venn diagram. Chao1 was used to estimate species richness, and Shannon's index was used to indicate species diversity. Sequences of 50 dominant bacterial OTUs were further compared to the nucleotide database using NCBI-BLAST tool, and the results are provided in Supplementary Table S2.

## 3. Results

### 3.1. Taxonomic Assignment of Reads

Metabarcoding analysis of 16S rRNA V3-V4 regions revealed a total of 156,865 raw sequences from four samples with the number of sequences ranging from 26,646 to 63,416 per individual sample (Table 1). After removing poor-quality reads, a total of 125,521 sequences were obtained. On removal of chimeras, 42,251, 25,893, 18,358, and 20,664 sequences were collected from dry zone, root zone, mineralized zone, and effluent water, respectively, resulting in 107,166 sequences from all samples. The total percentage of sequences flagged as chimeric was 14.6%.

### 3.2. Predominant Phyla and Genera

**Figure 3:**
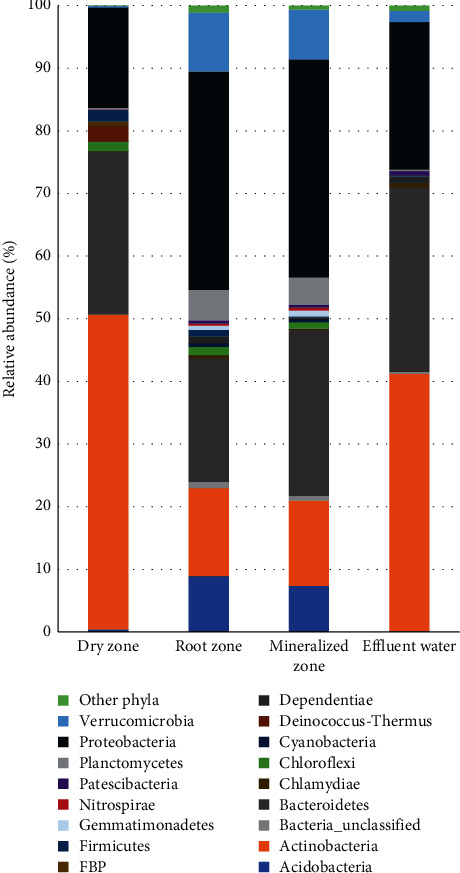
Classification of reads from dry zone, root zone, mineralized zone, and effluent water from the media grow bed at phylum level indicated as percentage of the population. Phyla that were represented in all zones below 0.5% of total community are displayed under “other phyla,” which contained the phyla Armatimonadetes, Cloacimonetes, Epsilonbacteraeota, Elusimicrobia, Fibrobacteres, Fusobacteria, Hydrogenedentes, Kiritimatiellaeota, Lentisphaerae, Omnitrophicaeota, Spirochaetes, Tenericutes, candidate division BRC1, WPS-2, and FCPU426.

Although the taxonomic composition of the bacterial community was similar between zones and effluent water, the frequency distribution of bacterial phyla differed between the three zones. All sequences were identified into 32 phyla, but only 17 had a relative abundance of more than 0.5% (Figure 3). The major phylum groups were Proteobacteria, Actinobacteria, and Bacteroidetes, accounting for more than 90% of the total community in the dry zone and effluent water. These phyla also accounted for more than 68% in the root and mineralized zones. Only a small fraction (0.02%–1.01%) of the total sequences for the samples could not be classified into any known phyla and were therefore labelled as unclassified sequence (Figure 3). The dry zone samples were mainly composed of Actinobacteria (50.28%), Bacteroidetes (26.04%), and Proteobacteria (16.09%). The root zone was mostly comprised of Proteobacteria (34.86%), Bacteroidetes (19.76%), and Actinobacteria (14.04%) while the mineralized zone was mostly comprised of Proteobacteria (34.82%), Bacteroidetes (26.49%), and Actinobacteria (13.60%). Effluent water was mainly characterized by Actinobacteria (41.10%), Bacteroidetes (29.50%), and Proteobacteria (23.54%).

Only 25 genera were dominant with a relative abundance of more than 1% of total bacteria reads (Table 2). The three media bed zones were dominated by different genera that included *Hymenobacter* (12.27%), *Massilia* (8.35%), *Pontibacter* (6.72%), and *Nocardioides* (6.66%) for the dry zone; *Mucilaginibacter* (3.17%) and *Rhodanobacter* (2.21%) for the root zone; and *Mizugakiibacter* (2.36%), *Heliimonas* (1.70%), and *Flavobacterium* (1.11%) for the mineralized zone (Table 2). The effluent water samples contained large numbers of *Rhodoluna* (26.64%), *Flavobacterium* (23.10%), and *Polynucleobacter* (10.47%).

### 3.3. Unique Bacterial Genera

Among the bacteria with an overall abundance of more than 1% of the total community, some unique genera were found in samples from media bed zones and effluent water (Table 3). Among them, 16 special genera were obtained in both the root zone and the mineralized zone, while ten unique genera were detected in the dry zone. Additionally, five unique genera were found in the effluent water. In the three zones, the main functions of the unique genera were divided into activities such as phosphorus solubilizing, production of antimicrobial substances, decomposition of organic matter, iron cycling, denitrification, nitrogen fixation, bioremediation, and production of secondary metabolites.

### 3.4. Bacterial Community Composition and Similarity Analysis

**Figure 4:**
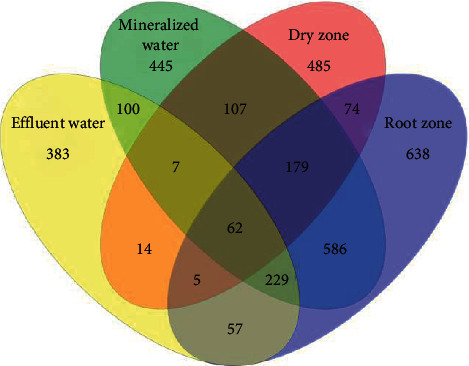
Venn diagram showing the unique and shared OTUs in samples from three media bed zones and the effluent water in an aquaponic system.

The number of shared OTUs for the three zones was 241 representing 7.15% of the total observed OTUs (3371) (Venn diagram, Figure 4). In addition, the unique OTU numbers in the grow bed samples from the dry zone, the root zone, the mineralized zone, and the effluent water were 485, 638, 445, and 383, respectively. The number of OTUs in the groups of the dry zone, root zone, mineralized zone, and effluent water was 933, 1,830, 1,715, and 857, respectively.

### 3.5. Diversity Analysis for Bacterial Communities

Each sample had coverage greater than 96% of Good's coverage, indicating that the sequencing depth was sufficient (Table 4). The Shannon index for the root zone was 6.26 suggesting a relatively higher diversity of bacterial sequences in this zone than in either the dry zone or mineralized zone. The community richness (total number of observed OTUs) and InvSimpson were highest in the root zone. Chao1 analysis conducted for estimating bacterial community richness indicated 1,489.80 phylotypes in the dry zone; 2,691.12 phylotypes in the root zone; 2,610.18 phylotypes in the mineralized zone; and 1,421.97 phylotypes in effluent water. Compared with the dry and the mineralized zones, the root zone had the highest OTU richness and bacterial community diversity.

Rarefaction curves were used to compare species richness in the three zones and effluent water (Supplementary Figure S1). The curves did not approach the asymptote, which indicated that each zone showed highest completeness of species. The curves indicated that OTUs were higher in samples from the root zone than the mineralized and dry zones (Supplementary Figure S1).

**Figure 5:**
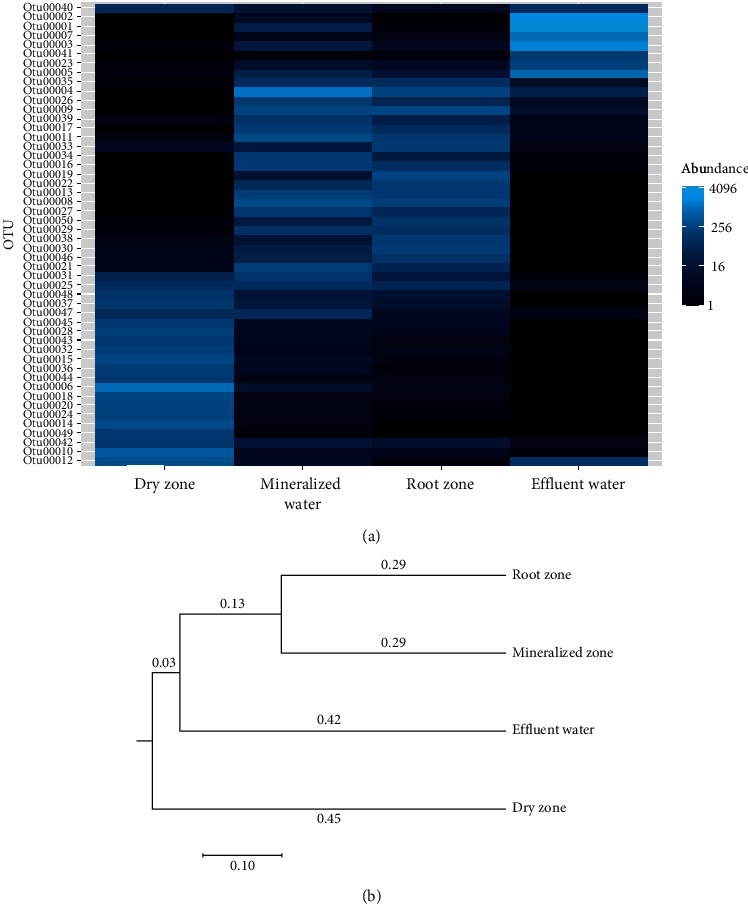
(a) A heat map showing the dominant OTUs and their relative abundance in the grow bed and effluent samples. (b) A dendrogram showing similarity of the aquaponic samples from three media bed zones and effluent water based on Jclass and Thetayc calculators in Mothur. Dominant bacterial OTUs are provided in Supplementary Table S1.

As shown in Figure 5(a), the abundances are exhibited in a heat map for the most abundant 50 OTUs. The pale light blue color illustrated highest abundance, and the black color expressed lowest abundance of species. The heat map indicates that most species in the root and mineralized zones had a higher relative abundance than the samples from the dry zone and effluent water. The dendrogram indicates that samples from root and mineralized zones had high similarity, while the dry zone was the most distantly related from all other zones including the effluent water (Figure 5(b)).

## 4. Discussion

### 4.1. The Bacterial Communities in the Vertical Zones and Effluent Water of a Flood-and-Drain Media Bed

#### 4.1.1. Dry Zone

The top 5–7 cm of the media grow bed was defined as the dry zone. This zone functions as a light barrier by preventing light from reaching the nutrient-rich water in the root zone. This zone protects light-sensitive beneficial bacteria and prevents algal growth [3]. The low moisture content in this zone reduces the growth of fungi and harmful bacteria. At the genus level, the genera *Pontibacter*, *Massilia*, *Modestobacter*, and *Hymenobacter* were the most dominant in the dry zone. Members of the genus *Massilia* have been reported in diverse environments [24]. This genus has been isolated from the rhizosphere and roots of plant species and appears to tolerate adverse environmental conditions [24]. *Massilia* utilizes root metabolites, degrades aromatic compounds, and produces antimicrobial substances [25, 26]. The reduced abundance of *Massilia* in other zones may be related to interspecies competition for resources, although this hypothesis needs to be tested in future studies. In addition, members of the genera *Pontibacter*, *Modestobacter*, and *Hymenobacter* are associated with harsh environmental conditions, including desert soils and surfaces of rocks [27, 28]. Thus, abundance of these genera in the dry zone may be related to the low water content and nutrient availability and high solar radiation.

#### 4.1.2. Root Zone

The root zone has a high moisture content. The depth for the root zone ranges from 10 to 25 cm [3]. Most of the biological activities occur in the root zone. The samples from the root zone contained high abundance of *Mucilaginibacter* and *Chujaibacter*. The genus *Mucilaginibacter* degrades polysaccharides using carbohydrate-active enzymes (CAZymes) [29]. They are regarded as cellulolytic bacteria that have an active role in plant biomass decomposition [30]. Thus, the dominance of *Mucilaginibacter* in this zone may be significant in the digestion and breakdown of organic matter and the mineralization process. The root zone also had a relatively high abundance of the putative denitrifiers *Rhodanobact*er and *Dokdonella*. This may be related to the setup of the flood-and-drain media growing technique. Media-based aquaponic systems may have pockets of anoxic zones that may provide favorable conditions for denitrification [31, 32]. The samples from the root zone had a relatively high abundance of *Acidibacter*, a ferric iron reducer [33]. Iron is an essential micronutrient for plant and fish growth in aquaponics [34]. It is typically available as soluble ferrous iron (Fe^2+^) and insoluble oxidized ferric iron (Fe^3+^). However, Fe^3+^ forms insoluble oxides and hydroxides, limiting its bioavailability [34]. Thus, the presence of *Acidibacter* may indicate Fe cycling in an aquaponic system. The aquaponic system in this study received Fe-EDDHA of 2 mg/L approximately every third week.

#### 4.1.3. Mineralized Zone

The bottom zone with an optimal depth range of 3 to 5 cm is responsible for the slow release of readily available nutrients into the system [3]. This zone includes mainly heterotrophic bacteria responsible for breaking down the waste into smaller molecules that can be absorbed by plants. The samples from the mineralized zone contained abundance of *Flavobacterium*, *Rurimicrobium*, *Rudaea*, *Mizugakiibacter*, and *Heliimonas*. *Rurimicrobium* and *Heliimonas* belong to *Chitinophagaceae* and are reported to produce antifungal metabolites [35]. *Mizugakiibacter* are heterotrophic bacteria typically found in the sediment of freshwater ecosystems [36]. Their high abundance in the mineralized zone might be caused by the solid wastes which tend to accumulate in this zone. *Flavobacterium* were more represented in samples from the mineralized zone than in the dry zone or root zone. *Flavobacterium* have been associated with the capacity to degrade complex organic compounds [37]. Under natural conditions, bacteria of the genus *Flavobacterium* mineralize organic substrates (e.g., carbohydrates, amino acids, and proteins) and degrade organic matter and some organisms (bacteria, fungi, and insects) using a variety of enzymes [37]. This genus is particularly concentrated in areas where solid waste accumulates [15]. The mineralized zone normally contains solid waste, and this may explain the relatively high abundance of *Flavobacterium* in this zone. Furthermore, the samples from the mineralized zone contained high abundance of *Rudaea*. The genus *Rudaea* belongs to the cellulolytic bacteria [38] and cellulose is mainly degraded in an aerobic environment. In soil-based studies, cellulolytic bacteria helped to regulate the carbon cycle through the decomposition of plant residues in the soil ecosystem [38, 39]. The relatively high abundance of *Rudaea* in this zone may be important for degrading organic matter and conversion of solid organic waste into useable nutrients for plants.

#### 4.1.4. Effluent Water

At genus level in the effluent water samples, *Rhodoluna*, *Flavobacterium*, *Polynucleobacter*, and *Aurantimicrobium* were dominant. These genera have been widely detected in freshwater environments [40–42]. Members of genus *Polynucleobacter* are capable of utilizing photo-oxidation products of humic substances, and some strains use light as an energy source [42]. Additionally, this bacterial group is considered as a good indicator for water quality through nitrogen and carbon cycling [41, 42]. Therefore, the presence of *Polynucleobacter* is a novel result because water quality variables are one of the main environmental considerations for optimizing aquaponic production [43]. The genera *Rhodoluna* and *Aurantimicrobium* are known as actinorhodopsin (ActR) encoding photoheterotrophic bacteria that can survive under low nutrient and energy conditions [44, 45]. ActR is a light-driven proton pump [44] and despite its presence in freshwater Actinobacteria, little is known about its ecological role including the range of wavelengths supporting the ActR proton pump activity [42, 46]. Therefore, the high abundance of *Rhodoluna* in effluent water may be related to ActR genes that enable this genus to successfully compete in a wide range of freshwater environments. In addition, the high abundance of the genus *Flavobacterium* in effluent water signifies degradation processes of organic matter in the flood-and-drain media bed.

### 4.2. Potential Influence of Bacteria on Media Bed Ecological Processes

Aquaponic gravel media beds are the habitat of a complex bacterial ecosystem. These microbial communities contribute to aquaponics through nitrification, nutrient conversion, regulation of organic matter decomposition, and mineralization of nutrients [10]. Such processes are vital for the functioning and health of aquaponic ecosystems. The present study explored the bacterial community structure of three vertically arranged zones of a flood-and-drain media bed from a mature coupled aquaponic system. The Shannon indices demonstrated that the bacterial richness in flood-and-drain media bed aquaponic system varies with depth. The root zone had the highest OTU richness and bacterial community diversity. The flood and drain action in the grow bed makes it highly aerobic, thereby providing a favorable environment for autotrophic and heterotrophic bacteria to form symbiotic relationships with the roots and perform critical functions of nitrification and mineralization, respectively. Additionally, root exudates released by plants might enhance microbial growth and distribution. For the mineralized zone, the design of the gravel bed in the flood-and-drain media bed aquaponic system ensures that mineral-rich water is being removed and replaced with new oxygenated water in this zone, providing conditions for conversion of solid wastes into useable nutrients and mineral elements for plants. The dry zone had the lowest bacterial community diversity. Being a unique zone that covers the root and mineralized zones from direct light and evaporation, it is likely that the conditions were characterized by relatively high temperature and direct sunlight, thus affecting the bacterial compositions [3].

Proteobacteria and Bacteroidetes were abundant across the zones and in the effluent water. These phyla respond rapidly to carbon sources and are considered as r-strategists, as they are fast-growing bacteria [16, 47]. The enrichment of Proteobacteria and Bacteroidetes in different units of an aquaponic system such as in the sump and biofilter was reported by Schmautz et al. [7] and Eck et al. [17], which is similar to the findings in our study. These phyla are mostly responsible for nitrogen cycling in aquaponic systems [7, 16]. Ammonium excreted by fish or derived from organic material is oxidized to nitrite and then nitrate by ammonia-oxidizing and nitrite-oxidizing bacteria belonging to the phylum Proteobacteria [3]. Under anoxic conditions, denitrifiers belonging to the phylum Bacteroidetes transform nitrate to nitrite, nitric oxide, nitrous oxide, and nitrogen gas [16].

A high proportion of reads from the dry zone was assigned to the phylum Actinobacteria. Actinobacteria is one of the largest phyla with members from a wide range of environmental sources, including soil surface [48]. They have been reported to be capable of producing a wide range of secondary metabolites [48, 49]. Actinobacteria produce extracellular hydrolytic enzymes, which degrade animal and plant residues and other organic compounds, allowing them to survive in environments with low nutrient levels [50, 51]. The presence of plant residues and probably the low nutrient levels in the dry zone might influence Actinobacteria to thrive in this type of environment. The relative abundance of Nitrospirae was relatively low, but samples from mineralized zones contained a higher percentage (0.5% of the total community) than the other zones.

## 5. Conclusions and Future Perspectives

The metabarcoding analysis presented here provides the first insight into the bacterial community of different zones of a flood-and-drain media bed system. The grow bed zones influenced the bacterial community structure. Specifically, the samples from the root zone harbored more diverse bacterial communities than the dry and mineralized zones. This could be due to similar conditions of nutrient-rich water that may increase niche overlap for more complex interactions between species. The findings were from samples collected from a single system at a single point in time; therefore, further research involving a more comprehensive sampling of a single system over time would be required to study the influence of sampling time on microbial diversity. A comparison between different media (e.g., pumice stones and expanded clay pebbles) and bacterial communities may yield insights into the effects of media on the bacterial ecology within this unique environment. Further studies to assess the relationship between different metabolites produced in different zones and bacterial community compositions are recommended.

## Figures and Tables

**Table 1:** Summary of metagenomic data for the vertical zones and samples from the effluent water.

Zone/source	Number of raw sequences	Number of sequences before chimeric	Number of sequences after chimeric	Chloroplast and mitochondria sequences	Number of reads after screening and filtering
Dry zone	63,416	49,368	42,251	111	41,886
Root zone	37,446	30,263	25,893	171	23,794
Mineralized zone	29,357	23,797	18,358	125	16,761
Effluent water	26,646	22,093	20,664	2	20,242

Total raw sequences: 156,865; total number of sequences before chimeras: 125,521; total number of sequences after removal of chimeras: 107,166.

**Table 2:** Distribution of different genera in the three media bed zones and effluent water.

Genera	Relative abundance (%)			
	Dry zone	Root zone	Mineralized zone	Effluent water
*Acidibacter*	0.00	2.04	0.71	0.00
*Actinomycetospora*	1.57	0.01	0.01	0.00
*Aeromicrobium*	2.31	0.03	0.05	0.00
*Blastococcus*	1.00	0.01	0.02	0.00
*Chujaibacter*	0.11	2.14	0.55	0.00
*Dokdonella*	0.00	0.67	1.34	0.02
*Flavobacterium*	0.21	0.89	1.11	23.10
*Flexivirga*	3.13	0.27	0.42	0.00
*Friedmanniella*	1.42	0.01	0.04	0.00
*Hymenobacter*	12.27	0.04	0.08	0.00
*Jatrophihabitans*	1.23	0.56	0.70	0.00
*Marmoricola*	3.14	0.17	0.35	0.00
*Massilia*	8.35	0.03	0.05	0.77
*Microbacterium*	2.14	0.70	0.85	0.01
*Mizugakiibacter*	0.01	1.26	2.36	0.03
*Modestobacter*	2.21	0.02	0.05	0.00
*Mucilaginibacter*	0.27	3.17	1.70	0.03
*Mycobacterium*	0.61	2.07	0.81	0.88
*Nocardioides*	6.66	0.36	0.98	0.00
*Opitutus*	0.00	0.41	1.14	0.14
*Pontibacter*	6.72	0.05	0.10	0.00
*Pseudonocardia*	1.71	0.04	0.13	0.00
*Rhodanobacter*	0.25	2.21	0.40	0.01
*Rhodoluna*	0.00	0.02	0.27	26.64
*Rudaea*	0.00	0.47	1.34	0.04

Only genera with relative abundance of ≥1% of the total community in at least one of the investigated samples are reported.

**Table 3:** Comparison of microbial genera among media bed zones and effluent water.

Genus	Function^*∗*^	Dry zone	Root zone	Mineralized zone	Effluent water
*Acidibacter*	Iron cycling	−	+	+	−
*Actinomycetospora*	Associative nitrogen fixation	+	+	+	−
*Aeromicrobium*	Nitrogen fixation	+	+	+	−
*Blastococcus*	Decomposition of organic matter	+	+	+	−
*Dokdonella*	Denitrification	−	+	+	+
*Flexivirga*	Degradation of organic matter	+	+	+	−
*Friedmanniella*	Production of antimicrobial substances	+	+	+	−
*Heliimonas*	Breakdown of complex organic compounds	−	+	+	+
*Jatrophihabitans*	Production of secondary metabolites	+	+	+	−
*Marmoricola*	Denitrification	+	+	+	−
*Modestobacter*	Phosphate solubilization	+	+	+	−
*Nocardioides*	Production of antibiotics, lignocellulose decomposition	+	+	+	−
*Opitutus*	Denitrification, polysaccharide degradation	−	+	+	+
*Pseudonocardia*	Nitrogen fixation	+	+	+	−
*Rhodoluna*	Ability to produce secondary metabolites that have antibiotic properties	−	+	+	+
*Rudaea*	Decomposition of plant residues	−	+	+	+
*Hymenobacter*	Decomposition of plant residues	+	+	+	−
*Pontibacter*	Nitrogen fixation	+	+	+	−

+: presence of the genus in the environment; −: absence of the genus in the environment. ^*∗*^The function description of each genus is based on Taxonomic Outline of the Prokaryotes, Bergey's Manual of Systematic Bacteriology by Holt et al. [22], and the Prokaryotes by Dworkin et al. [23].

**Table 4:** Richness and diversity indices of bacterial communities for aquaponic samples collected from three media bed zones and effluent water.

Zone	Chao1	InvSimpson	Shannon	Good's coverage (%)	Observed richness
Dry zone	1,489.80	80.69	5.24	98	933
Root zone	2,691.12	218.89	6.26	96	1,830
Mineralized zone	2,610.18	93.21	5.89	96	1,715
Effluent water	1,421.97	7.33	3.21	98	857

## Data Availability

The sequence datasets generated in this study have been deposited in the sequence reads archive (SRA) database of the National Center of Biotechnology Information (SRA accession: SRR11855284).

## Supplementary Materials

Supplementary Materials Table S1: taxonomic classification of the 50 most abundant OTUs. The taxonomical classification was performed to genus level. The dominant bacterial OTUs are provided in the table. Table S2: bacterial OTUs and their most related species. Sequences of 50 dominant bacterial OTUs were further compared to the nucleotide database using NCBI-BLAST tool, and the results are provided in the table. Figure S1: rarefaction analysis of samples from three grow bed zones and the effluent water in an aquaponic system. Rarefaction curves were used to compare species richness in the three zones and effluent water.
