# Supplementary material for: Metabarcoding Analysis of Bacterial Communities Associated with Media Grow Bed Zones in an Aquaponic System
Source: Int J Microbiol. 2020 Oct 1;2020:8884070. doi: 10.1155/2020/8884070 (PMC7547338; doi:10.1155/2020/8884070)
Supplement: Supplementary Materials — Table S1: taxonomic classification of the 50 most abundant OTUs. The taxonomical classification was performed to genus level. The dominant bacterial OTUs are provided in the table. Table S2: bacterial OTUs and their most related species. Sequences of 50 dominant bacterial OTUs were further compared to the nucleotide database using NCBI-BLAST tool, and the results are provided in the table. Figure S1: rarefaction analysis of samples from three grow bed zones and the effluent water in an aquaponic system. Rarefaction curves were used to compare species richness in the three zones and effluent water. [file 8884070.f1.docx]

**Supplementary Material**

TABLE S1: Taxonomic classification of the 50 most abundant OTUs.

TABLE S2: Bacterial OTUs and their most related species.

FIGURE S1: Rarefaction analysis of samples from three grow bed zones and the effluent water in an aquaponics system.

TABLE S1: Taxonomic classification of the 50 most abundant OTUs

| OTU | Taxonomy |
| --- | --- |
| Otu00001 | Actinobacteria(100); Micrococcales(100);Microbacteriaceae(100);Microbacteriaceae_unclassified(100); |
| Otu00002 | Bacteroidetes(100); Flavobacteriales(100);Flavobacteriaceae(100);Flavobacterium(100); |
| Otu00003 | Proteobacteria(100); Betaproteobacteriales(100);Burkholderiaceae(100);Polynucleobacter(100); |
| Otu00004 | Bacteroidetes(100); Chitinophagales(100);Chitinophagaceae(100);uncultured(99); |
| Otu00005 | Actinobacteria(100); Micrococcales(100);Microbacteriaceae(100);Microbacteriaceae_unclassified(100); |
| Otu00006 | Bacteroidetes(100); Cytophagales(100);Hymenobacteraceae(100);Pontibacter(100); |
| Otu00007 | Actinobacteria(100); Micrococcales(100);Microbacteriaceae(100);Aurantimicrobium(100); |
| Otu00008 | Proteobacteria(100); Xanthomonadales(100);Xanthomonadales_unclassified(97);Xanthomonadales_unclassified(97); |
| Otu00009 | Bacteroidetes(100); Cytophagales(100);Microscillaceae(100);uncultured(100); |
| Otu00010 | Proteobacteria(100); Betaproteobacteriales(100);Burkholderiaceae(100);Massilia(100); |
| Otu00011 | Proteobacteria(100); Xanthomonadales(100);Rhodanobacteraceae(100);Mizugakiibacter(100); |
| Otu00012 | Proteobacteria(100); Betaproteobacteriales(100);Burkholderiaceae(100);Massilia(99); |
| Otu00013 | Bacteroidetes(100); Chitinophagales(100);Chitinophagaceae(100);Heliimonas(100); |
| Otu00014 | Proteobacteria(100); Betaproteobacteriales(100);Burkholderiaceae(100);Massilia(100); |
| Otu00015 | Actinobacteria(100); Frankiales(100);Geodermatophilaceae(100);Modestobacter(100); |
| Otu00016 | Bacteroidetes(100); Chitinophagales(100);Chitinophagaceae(100);Chitinophagaceae_unclassified(100); |
| Otu00017 | Proteobacteria(100); Xanthomonadales(100);Rhodanobacteraceae(98);Dokdonella(97); |
| Otu00018 | Bacteroidetes(100); Cytophagales(100);Hymenobacteraceae(100);Hymenobacter(100); |
| Otu00019 | Proteobacteria(100); Gammaproteobacteria_Incertae_Sedis(97);Unknown_Family(97);Acidibacter(97); |
| Otu00020 | Bacteroidetes(100); Cytophagales(100);Hymenobacteraceae(100);Hymenobacter(100); |
| Otu00021 | Actinobacteria(100); Actinobacteria_unclassified(65);Actinobacteria_unclassified(65);Actinobacteria_unclassified(65); |
| Otu00022 | Proteobacteria(100); Xanthomonadales(100);Xanthomonadaceae(100);Xanthomonadaceae_unclassified(100); |
| Otu00023 | Bacteroidetes(100); Flavobacteriales(100);Flavobacteriaceae(100);Flavobacterium(100); |
| Otu00024 | Deinococcus-Thermus(100); Deinococcales(100);Deinococcaceae(100);Deinococcus(100); |
| Otu00025 | Actinobacteria(100);Micrococcales(100);Microbacteriaceae(100);Microbacterium(53); |
| Otu00026 | Proteobacteria(100); Xanthomonadales(100);Rhodanobacteraceae(100);Rudaea(100); |
| Otu00027 | Acidobacteria(100); Subgroup_2(100);Subgroup_2_fa(100);Subgroup_2_ge(100); |
| Otu00028 | Actinobacteria(100); Propionibacteriales(100);Nocardioidaceae(100);Aeromicrobium(100); |
| Otu00029 | Bacteroidetes(100); Sphingobacteriales(100);Sphingobacteriaceae(100);Mucilaginibacter(100); |
| Otu00030 | Proteobacteria(100); Xanthomonadales(100);Rhodanobacteraceae(100);Chujaibacter(100); |
| Otu00031 | Actinobacteria(100); Micrococcales(100);Intrasporangiaceae(100);Intrasporangiaceae_unclassified(100); |
| Otu00032 | Actinobacteria(100); Micrococcales(100);Intrasporangiaceae(100);Janibacter(67); |
| Otu00033 | Proteobacteria(100); Xanthomonadales(100);Rhodanobacteraceae(100);Rhodanobacter(99); |
| Otu00034 | Proteobacteria(100); Steroidobacterales(98);Steroidobacteraceae(98);uncultured(98); |
| Otu00035 | Proteobacteria(100); Betaproteobacteriales(100);Burkholderiaceae(100);Burkholderiaceae_unclassified(100); |
| Otu00036 | Actinobacteria(100); Propionibacteriales(100);Propionibacteriaceae(100);Friedmanniella(100); |
| Otu00037 | Actinobacteria(100); Micrococcales(100);Micrococcales_unclassified(97);Micrococcales_unclassified(97); |
| Otu00038 | Verrucomicrobia(100); Opitutales(100);Opitutaceae(100);Opitutaceae_unclassified(99); |
| Otu00039 | Proteobacteria(100); Betaproteobacteriales(100);Burkholderiaceae(100);Burkholderiaceae_unclassified(91); |
| Otu00040 | Proteobacteria(100); Betaproteobacteriales(100);Burkholderiaceae(100);Burkholderiaceae_unclassified(88); |
| Otu00041 | Proteobacteria(100); Betaproteobacteriales(100);Burkholderiaceae(100);Burkholderiaceae_unclassified(98); |
| Otu00042 | Actinobacteria(100); Micrococcales(100);Microbacteriaceae(100);Microbacteriaceae_unclassified(59); |
| Otu00043 | Actinobacteria(100); Micrococcales(100);Micrococcaceae(100);Pseudarthrobacter(64); |
| Otu00044 | Bacteroidetes(100);Cytophagales(100); Hymenobacteraceae(100);Hymenobacter(100); |
| Otu00045 | Actinobacteria(100); Pseudonocardiales(100);Pseudonocardiaceae(100);Pseudonocardia(100); |
| Otu00046 | Proteobacteria(100); Betaproteobacteriales(100);Burkholderiaceae(100);Burkholderia-Caballeronia-Paraburkholderia(99); |
| Otu00047 | Bacteroidetes(100); Flavobacteriales(100);Weeksellaceae(100);Bergeyella(100); |
| Otu00048 | Actinobacteria(100); Micrococcales(100);Dermacoccaceae(100);Flexivirga(100); |
| Otu00049 | Bacteroidetes(100); Cytophagales(100);Hymenobacteraceae(100);Hymenobacter(100); |
| Otu00050 | Bacteroidetes(100); Chitinophagales(100);Chitinophagaceae(100);uncultured(74); |

TABLE S2*:* Bacterial OTUs and their most related species.

| Bacterial OTU | Related species (NCBI-Blast) | Similarity (%) | Accession | Phylum |
| --- | --- | --- | --- | --- |
| Otu00001 | [*Candidatus* Rhodoluna planktonica](https://blast.ncbi.nlm.nih.gov/Blast.cgi#alnHdr_672238899) | 97.23% | [NR_125488.1](https://www.ncbi.nlm.nih.gov/nucleotide/NR_125488.1?report=genbank&log$=nucltop&blast_rank=1&RID=KJ9T3P5J014) | Actinobacteria |
| Otu00002 | [*Flavobacterium keumense* strain K3R-10](https://blast.ncbi.nlm.nih.gov/Blast.cgi#alnHdr_1441204206) | 98.96% | [NR_157621.1](https://www.ncbi.nlm.nih.gov/nucleotide/NR_157621.1?report=genbank&log$=nucltop&blast_rank=1&RID=KJ9Z0PHE014) | Bacteroidetes |
| Otu00003 | [*Polynucleobacter aenigmaticus* strain MWH-K35W1](https://blast.ncbi.nlm.nih.gov/Blast.cgi#alnHdr_1491505232) | 98.27% | [NR_159080.1](https://www.ncbi.nlm.nih.gov/nucleotide/NR_159080.1?report=genbank&log$=nucltop&blast_rank=1&RID=KJA3MCHB016) | Proteobacteria |
| Otu00004 | [*Rurimicrobium arvi* strain J107-1](https://blast.ncbi.nlm.nih.gov/Blast.cgi#alnHdr_1491515806) | 94.12% | [NR_159218.1](https://www.ncbi.nlm.nih.gov/nucleotide/NR_159218.1?report=genbank&log$=nucltop&blast_rank=1&RID=KJA5XUEJ016) | Bacteroidetes |
| Otu00005 | [*Leifsonia lichenia* strain 2Sb](https://blast.ncbi.nlm.nih.gov/Blast.cgi#alnHdr_631251447) | 97.23% | [NR_112644.1](https://www.ncbi.nlm.nih.gov/nucleotide/NR_112644.1?report=genbank&log$=nucltop&blast_rank=1&RID=KJA9SF3C014) | Actinobacteria |
| Otu00006 | [*Pontibacter diazotrophicus* strain H4X](https://blast.ncbi.nlm.nih.gov/Blast.cgi#alnHdr_699005419) | 97.23% | [NR_126288.1](https://www.ncbi.nlm.nih.gov/nucleotide/NR_126288.1?report=genbank&log$=nucltop&blast_rank=1&RID=KJAE0HJA014) | Bacteroidetes |
| Otu00007 | [*Aurantimicrobium minutum* strain KNC](https://blast.ncbi.nlm.nih.gov/Blast.cgi#alnHdr_1137647826) | 98.62% | [NR_145615.1](https://www.ncbi.nlm.nih.gov/nucleotide/NR_145615.1?report=genbank&log$=nucltop&blast_rank=1&RID=KJAGPN65014) | Actinobacteria |
| Otu00008 | [*Rudaea cellulosilytica* strain KIS3-4](https://blast.ncbi.nlm.nih.gov/Blast.cgi#alnHdr_343206014) | 96.19% | [NR_044566.1](https://www.ncbi.nlm.nih.gov/nucleotide/NR_044566.1?report=genbank&log$=nucltop&blast_rank=1&RID=KJAJRC66014) | Proteobacteria |
| Otu00009 | [*Chryseolinea soli* strain KIS68-18](https://blast.ncbi.nlm.nih.gov/Blast.cgi#alnHdr_1811131526) | 86.55% | [NR_165708.1](https://www.ncbi.nlm.nih.gov/nucleotide/NR_165708.1?report=genbank&log$=nucltop&blast_rank=1&RID=KJAPMRVF016) | Bacteroidetes |
| Otu00010 | [*Massilia psychrophil*a strain B1555-1](https://blast.ncbi.nlm.nih.gov/Blast.cgi#alnHdr_1277396367) | 100.00% | [NR_152080.1](https://www.ncbi.nlm.nih.gov/nucleotide/NR_152080.1?report=genbank&log$=nucltop&blast_rank=1&RID=KJAV1W1P016) | Proteobacteria |
| Otu00011 | [*Mizugakiibacter sediminis* strain skMP5](https://blast.ncbi.nlm.nih.gov/Blast.cgi#alnHdr_699005327) | 95.85% | [NR_126196.1](https://www.ncbi.nlm.nih.gov/nucleotide/NR_126196.1?report=genbank&log$=nucltop&blast_rank=1&RID=KJAX30W0014) | Proteobacteria |
| Otu00012 | [*Massilia pinisoli* strain T33](https://blast.ncbi.nlm.nih.gov/Blast.cgi#alnHdr_1277396298) | 98.96% | [NR_152009.1](https://www.ncbi.nlm.nih.gov/nucleotide/NR_152009.1?report=genbank&log$=nucltop&blast_rank=1&RID=KJAZEES6016) | Proteobacteria |
| Otu00013 | [*Heliimonas saccharivorans* strain L2-4](https://blast.ncbi.nlm.nih.gov/Blast.cgi#alnHdr_1011034795) | 95.50% | [NR_135701.1](https://www.ncbi.nlm.nih.gov/nucleotide/NR_135701.1?report=genbank&log$=nucltop&blast_rank=5&RID=KJB6HMBT01R) | Bacteroidetes |
| Otu00014 | *Massilia agri* strain K-3-1 | 98.96% | [NR_157781.1](https://www.ncbi.nlm.nih.gov/nucleotide/NR_157781.1?report=genbank&log$=nucltop&blast_rank=1&RID=KJB8J4V901R) | Proteobacteria |
| Otu00015 | [*Modestobacter caceresii* strain KNN 45-2b](https://blast.ncbi.nlm.nih.gov/Blast.cgi#alnHdr_1040567049) | 98.62% | [NR_137398.1](https://www.ncbi.nlm.nih.gov/nucleotide/NR_137398.1?report=genbank&log$=nucltop&blast_rank=1&RID=KJBBPAKD014) | Actinobacteria |
| Otu00016 | [*Sediminibacterium aquarii* strain AA5](https://blast.ncbi.nlm.nih.gov/Blast.cgi#alnHdr_1315203998) | 94.46% | [NR_152667.1](https://www.ncbi.nlm.nih.gov/nucleotide/NR_152667.1?report=genbank&log$=nucltop&blast_rank=1&RID=KJBD5FWY014) | Bacteroidetes |
| Otu00017 | *Dokdonella fugitiva* strain A3 | 97.58% | [NR_042397.1](https://www.ncbi.nlm.nih.gov/nucleotide/NR_042397.1?report=genbank&log$=nucltop&blast_rank=1&RID=KJBH1N1C016) | Proteobacteria |
| Otu00018 | [*Hymenobacter gummosu*s strain ANT-18](https://blast.ncbi.nlm.nih.gov/Blast.cgi#alnHdr_1491509601) | 99.31% | [NR_159132.1](https://www.ncbi.nlm.nih.gov/nucleotide/NR_159132.1?report=genbank&log$=nucltop&blast_rank=2&RID=KJBK3CMV01R) | Bacteroidetes |
| Otu00019 | [*Acidibacter ferrireducens* strain MCF85](https://blast.ncbi.nlm.nih.gov/Blast.cgi#alnHdr_699005391) | 100.00% | [NR_126260.1](https://www.ncbi.nlm.nih.gov/nucleotide/NR_126260.1?report=genbank&log$=nucltop&blast_rank=1&RID=KJBNKUBC014) | Proteobacteria |
| Otu00020 | *H*[*ymenobacter profundi* strain M2](https://blast.ncbi.nlm.nih.gov/Blast.cgi#alnHdr_1520188720) | 98.62% | [NR_159924.1](https://www.ncbi.nlm.nih.gov/nucleotide/NR_159924.1?report=genbank&log$=nucltop&blast_rank=1&RID=KJBTPBFB014) | Bacteroidetes |
| Otu00021 | [*Angustibacter aerolatus* strain 7402J-48](https://blast.ncbi.nlm.nih.gov/Blast.cgi#alnHdr_566085541) | 97.92% | [NR_109610.1](https://www.ncbi.nlm.nih.gov/nucleotide/NR_109610.1?report=genbank&log$=nucltop&blast_rank=1&RID=KJBX4167014) | Actinobacteria |
| Otu00022 | [*Luteimonas soli* strain Y2](https://blast.ncbi.nlm.nih.gov/Blast.cgi#alnHdr_1146059181) | 94.46% | [NR_145913.1](https://www.ncbi.nlm.nih.gov/nucleotide/NR_145913.1?report=genbank&log$=nucltop&blast_rank=1&RID=KJC17GY0014) | Proteobacteria |
| Otu00023 | [*Flavobacterium fluviatile* strain TAPY14](https://blast.ncbi.nlm.nih.gov/Blast.cgi#alnHdr_1679775908) | 100.00% | [NR_163630.1](https://www.ncbi.nlm.nih.gov/nucleotide/NR_163630.1?report=genbank&log$=nucltop&blast_rank=1&RID=KJC5198J014) | Bacteroidetes |
| Otu00024 | *Deinococcus swuensis* strain DY59 | 98.27% | [NR_132693.1](https://www.ncbi.nlm.nih.gov/nucleotide/NR_132693.1?report=genbank&log$=nucltop&blast_rank=1&RID=KJC8714G016) | Deinococcus-Thermus |
| Otu00025 | [*Microbacterium paludicola* strain US15](https://blast.ncbi.nlm.nih.gov/Blast.cgi#alnHdr_636558882) | 99.65% | [NR_114939.1](https://www.ncbi.nlm.nih.gov/nucleotide/NR_114939.1?report=genbank&log$=nucltop&blast_rank=1&RID=KJC9X29H014) | Actinobacteria |
| Otu00026 | [*Rudaea cellulosilytica* strain KIS3-4](https://blast.ncbi.nlm.nih.gov/Blast.cgi#alnHdr_343206014) | 98.27% | [NR_044566.1](https://www.ncbi.nlm.nih.gov/nucleotide/NR_044566.1?report=genbank&log$=nucltop&blast_rank=1&RID=KJCCT3YW014) | Proteobacteria |
| Otu00027 | [*Edaphobacter dinghuensis* strain DHF9](https://blast.ncbi.nlm.nih.gov/Blast.cgi#alnHdr_1199303397) | 87.89% | [NR_147748.1](https://www.ncbi.nlm.nih.gov/nucleotide/NR_147748.1?report=genbank&log$=nucltop&blast_rank=1&RID=KJCJDY9V014) | Acidobacteria |
| Otu00028 | [*Aeromicrobium fastidiosum* strain DSM 10552](https://blast.ncbi.nlm.nih.gov/Blast.cgi#alnHdr_645322654) | 98.27% | [NR_119352.1](https://www.ncbi.nlm.nih.gov/nucleotide/NR_119352.1?report=genbank&log$=nucltop&blast_rank=1&RID=KJCP9T5N014) | Actinobacteria |
| Otu00029 | [*Mucilaginibacter puniceus* strain WS71](https://blast.ncbi.nlm.nih.gov/Blast.cgi#alnHdr_1315203999) | 97.58% | [NR_152668.1](https://www.ncbi.nlm.nih.gov/nucleotide/NR_152668.1?report=genbank&log$=nucltop&blast_rank=1&RID=KJCUF5Y9014) | Bacteroidetes |
| Otu00030 | [*Chujaibacter soli* strain KIS55-21](https://blast.ncbi.nlm.nih.gov/Blast.cgi#alnHdr_1137647757) | 97.58% | [NR_145539.1](https://www.ncbi.nlm.nih.gov/nucleotide/NR_145539.1?report=genbank&log$=nucltop&blast_rank=1&RID=KJCWP2B6014) | Proteobacteria |
| Otu00031 | [*Phycicoccus jejuensi*s strain KSW2-15](https://blast.ncbi.nlm.nih.gov/Blast.cgi#alnHdr_636559688) | 98.96% | [NR_115748.1](https://www.ncbi.nlm.nih.gov/nucleotide/NR_115748.1?report=genbank&log$=nucltop&blast_rank=1&RID=KJCYMSVH014) | Actinobacteria |
| Otu00032 | *J*[*anibacter cremeus* strain HR08-44](https://blast.ncbi.nlm.nih.gov/Blast.cgi#alnHdr_631253182) | 99.31% | [NR_114380.1](https://www.ncbi.nlm.nih.gov/nucleotide/NR_114380.1?report=genbank&log$=nucltop&blast_rank=1&RID=KJD8AMG9014) | Actinobacteria |
| Otu00033 | [*Rhodanobacter xiangquanii* strain BJQ-6](https://blast.ncbi.nlm.nih.gov/Blast.cgi#alnHdr_926663116) | 98.62% | [NR_132710.1](https://www.ncbi.nlm.nih.gov/nucleotide/NR_132710.1?report=genbank&log$=nucltop&blast_rank=1&RID=KJDAJUE7014) | Proteobacteria |
| Otu00034 | [*Immundisolibacter cernigliae* strain TR3.2](https://blast.ncbi.nlm.nih.gov/Blast.cgi#alnHdr_1397641697) | 92.39% | [NR_156801.1](https://www.ncbi.nlm.nih.gov/nucleotide/NR_156801.1?report=genbank&log$=nucltop&blast_rank=1&RID=KJDC9RU6016) | Proteobacteria |
| Otu00035 | [*Rhodoferax antarcticus* ANT.BR](https://blast.ncbi.nlm.nih.gov/Blast.cgi#alnHdr_559795245) | 98.96% | [NR_104835.1](https://www.ncbi.nlm.nih.gov/nucleotide/NR_104835.1?report=genbank&log$=nucltop&blast_rank=1&RID=KJDJ1THW014) | Proteobacteria |
| Otu00036 | [*Friedmanniella spumicola* strain Ben 107](https://blast.ncbi.nlm.nih.gov/Blast.cgi#alnHdr_219857276) | 99.31% | [NR_024907.1](https://www.ncbi.nlm.nih.gov/nucleotide/NR_024907.1?report=genbank&log$=nucltop&blast_rank=1&RID=KJDN2VD8016) | Actinobacteria |
| Otu00037 | [*Flexivirga lutea* strain TBS-100](https://blast.ncbi.nlm.nih.gov/Blast.cgi#alnHdr_1277396239) | 99.31% | [NR_151950.1](https://www.ncbi.nlm.nih.gov/nucleotide/NR_151950.1?report=genbank&log$=nucltop&blast_rank=1&RID=KJDPKBW9016) | Actinobacteria |
| Otu00038 | [*Opitutus terrae* PB90-1](https://blast.ncbi.nlm.nih.gov/Blast.cgi#alnHdr_265678585) | 94.83% | [NR_028890.1](https://www.ncbi.nlm.nih.gov/nucleotide/NR_028890.1?report=genbank&log$=nucltop&blast_rank=2&RID=KJDSNKC5016) | Verrucomicrobia |
| Otu00039 | [*Ramlibacter alkalitolerans* strain CJ661](https://blast.ncbi.nlm.nih.gov/Blast.cgi#alnHdr_1491514564) | 99.65% | [NR_159174.1](https://www.ncbi.nlm.nih.gov/nucleotide/NR_159174.1?report=genbank&log$=nucltop&blast_rank=1&RID=KJDW0H0V016) | Proteobacteria |
| Otu00040 | [*Acidovorax caeni* strain R-24608](https://blast.ncbi.nlm.nih.gov/Blast.cgi#alnHdr_343201701) | 99.65% | [NR_042427.1](https://www.ncbi.nlm.nih.gov/nucleotide/NR_042427.1?report=genbank&log$=nucltop&blast_rank=1&RID=KJDYSRDC016) | Proteobacteria |
| Otu00041 | [*Orrella dioscoreae* strain LMG 29303](https://blast.ncbi.nlm.nih.gov/Blast.cgi#alnHdr_1558439486) | 97.92% | [NR_160523.1](https://www.ncbi.nlm.nih.gov/nucleotide/NR_160523.1?report=genbank&log$=nucltop&blast_rank=1&RID=KJE181X8014) | Proteobacteria |
| Otu00042 | [*Chryseoglobus frigidaquae* strain CW1](https://blast.ncbi.nlm.nih.gov/Blast.cgi#alnHdr_636559939) | 99.65% | [NR_115999.1](https://www.ncbi.nlm.nih.gov/nucleotide/NR_115999.1?report=genbank&log$=nucltop&blast_rank=1&RID=KJE5E8Y2016) | Actinobacteria |
| Otu00043 | *Pseudarthrobacter scleromae* strain YH-2001 | 98.27% | NR_041824.1 | Actinobacteria |
| Otu00044 | [*Hymenobacter rigui* strain NBRC 101118](https://blast.ncbi.nlm.nih.gov/Blast.cgi#alnHdr_631252781) | 98.62% | [NR_113979.1](https://www.ncbi.nlm.nih.gov/nucleotide/NR_113979.1?report=genbank&log$=nucltop&blast_rank=1&RID=KJEENNK8014) | Bacteroidetes |
| Otu00045 | [*Pseudonocardia xishanensis* strain YIM 63638](https://blast.ncbi.nlm.nih.gov/Blast.cgi#alnHdr_1270533043) | 99.66% | [NR_108411.2](https://www.ncbi.nlm.nih.gov/nucleotide/NR_108411.2?report=genbank&log$=nucltop&blast_rank=1&RID=KJEGYAGF014) | Actinobacteria |
| Otu00046 | [*Paraburkholderia metalliresistens* strain D414](https://blast.ncbi.nlm.nih.gov/Blast.cgi#alnHdr_1024974858) | 100.00% | [NR_136833.1](https://www.ncbi.nlm.nih.gov/nucleotide/NR_136833.1?report=genbank&log$=nucltop&blast_rank=1&RID=KJEMCYEH014) | Proteobacteria |
| Otu00047 | *Bergeyella zoohelcum* strain D658 | 92.73% | NR_104718.1 | Bacteroidetes |
| Otu00048 | [*Flexivirga lutea* strain TBS-100](https://blast.ncbi.nlm.nih.gov/Blast.cgi#alnHdr_1277396239) | 98.62% | [NR_151950.1](https://www.ncbi.nlm.nih.gov/nucleotide/NR_151950.1?report=genbank&log$=nucltop&blast_rank=1&RID=KJES9G3W014) | Actinobacteria |
| Otu00049 | *Hymenobacter rivuli* strain TAPP3 | 99.31% | [NR_163628.1](https://www.ncbi.nlm.nih.gov/nucleotide/NR_163628.1?report=genbank&log$=nucltop&blast_rank=1&RID=KJEU2J0U016) | Bacteroidetes |
| Otu00050 | [*Puia dinghuensis* strain 4GSH07](https://blast.ncbi.nlm.nih.gov/Blast.cgi#alnHdr_1491509597) | 97.58% | [NR_159128.1](https://www.ncbi.nlm.nih.gov/nucleotide/NR_159128.1?report=genbank&log$=nucltop&blast_rank=1&RID=KJEW6BGP016) | Bacteroidetes |

FIGURE 1S: Rarefaction analysis of samples from three grow bed zones and the effluent water in an aquaponics system. Rarefaction curves of OTUs clustered at 97% sequence identity among samples.
